# On the Frequent Occurrence of Mitigated Variola after Vaccination

**Published:** 1815-12

**Authors:** 


*For the London Medical and Physical Journal.*

### *On the frequent Occurrence of Mitigated Variola after Vaccination;* by MEDICUS.

IF the following case be considered worthy of notice, I will thank you to insert it, as preparatory to some remarks I have to make on the subject of vaccination.

L. Downs, aged seven, was vaccinated at the Pottery Dispensary six years ago. Her mother can scarcely recollect whether the virus exerted its proper influence on the system. Some time since she sickened, and at the usual period the small-pox appeared. The eruptive fever was very considerable, but she went through the course of the disease without any bad symptom, and quickly recovered. The pustules were larger, full of matter, and much dispersed over the face and trunk. She is one of eleven children, who were constantly with her, and many of them nursing her: they have been all vaccinated, and they have escaped the infection. It is also worthy of remark, that a very malignant species was raging at the next house, from whence I have no doubt that she caught her disorder, and that its favourable progress was attributable to her previous vaccination.

I am sorry to say that this is by no means the only case of variola appearing after vaccination in this part of the world. On the contrary, the instances are sufficiently numerous to have created a considerable reluctance on the part of parents to submit their children to it; and hence the small-pox has been very rife during the last summer: it has, however, been generally favourable, the malignant species only occurring where children were placed in small heated rooms, and stimulants improperly administered.

For the present, I only wish to propose two questions with regard regard to vaccine virus, begging leave to return to the subject at another opportunity.

1. Is not the efficacy of the vaccine virus diminished by its being constantly passed through the human system?

2. Would it not be advantageous to have a more frequent recourse to the fountain of the disease?

*Newcastle, Staffordshire.*

