# Erratum

**Published:** 1816-08

**Authors:** 


In a former communication, in vol. xxxiv. page 454, line fifth, for *conclusion* read *non-existence.*

